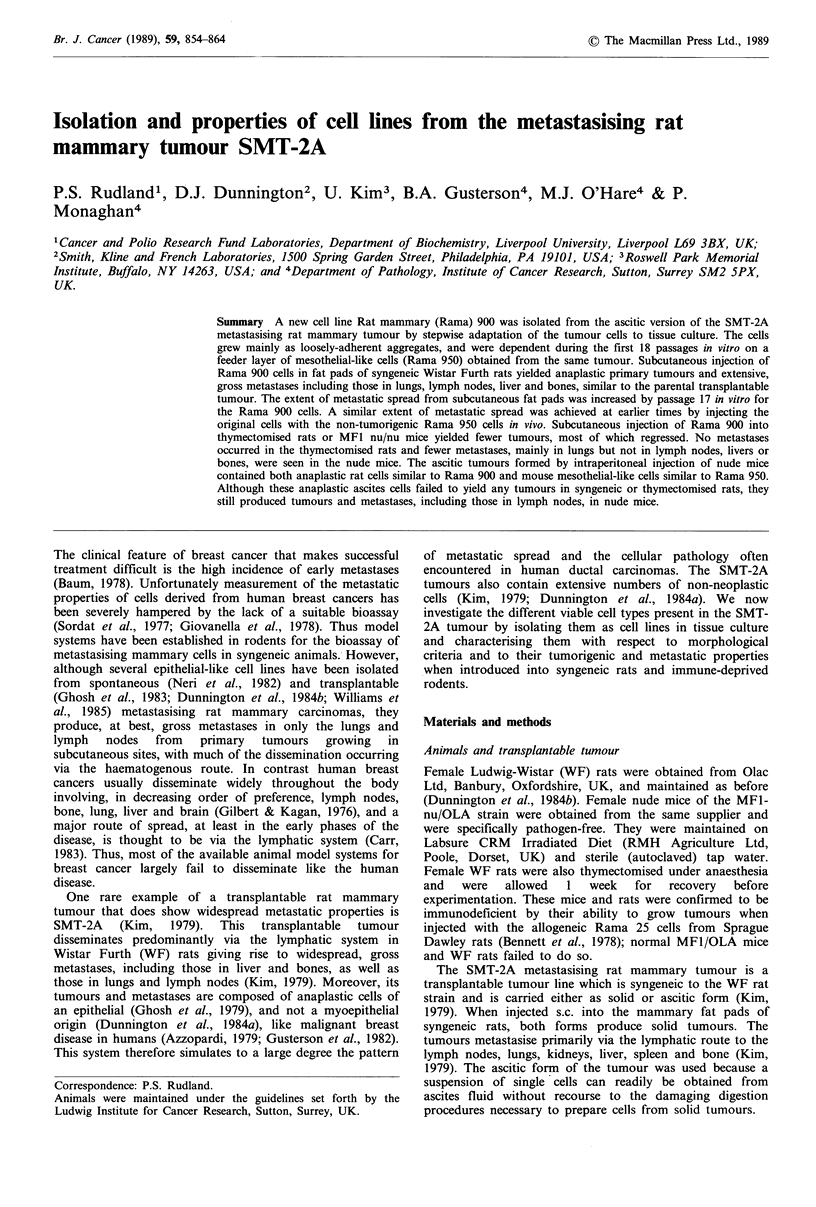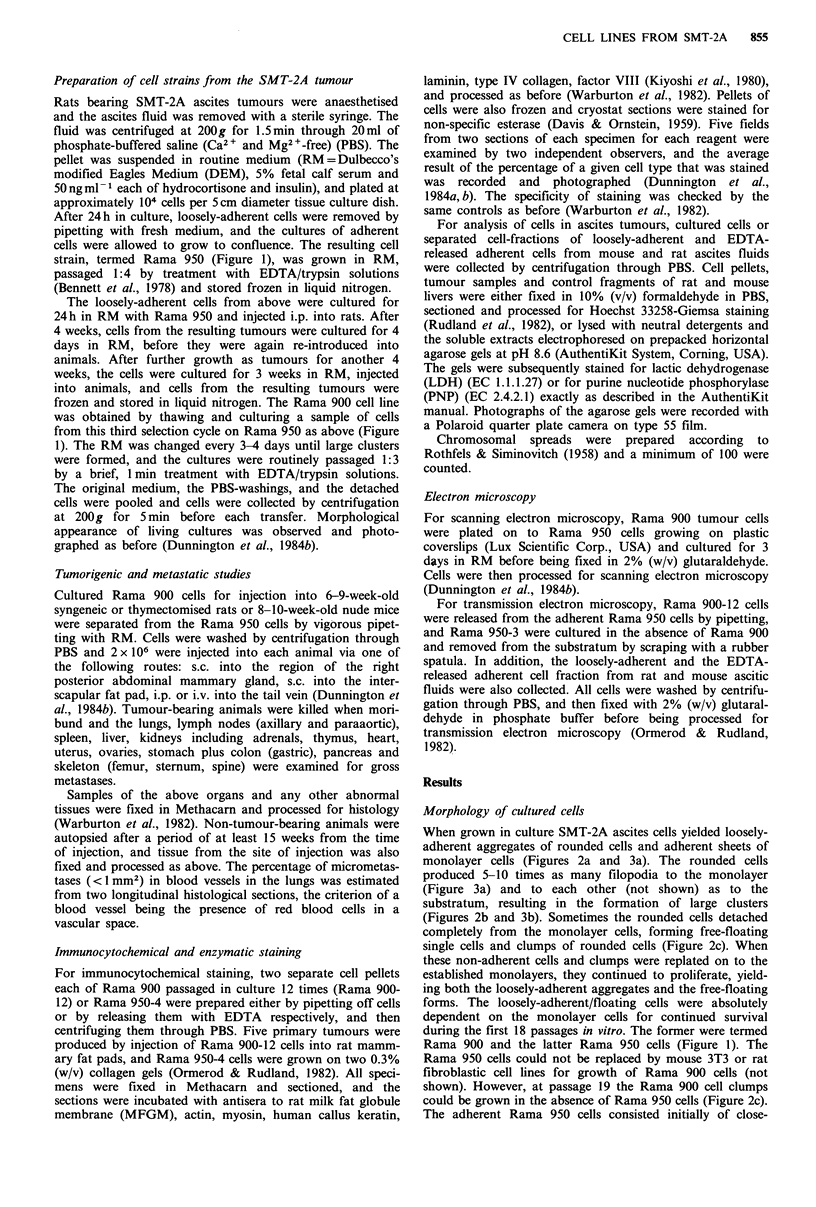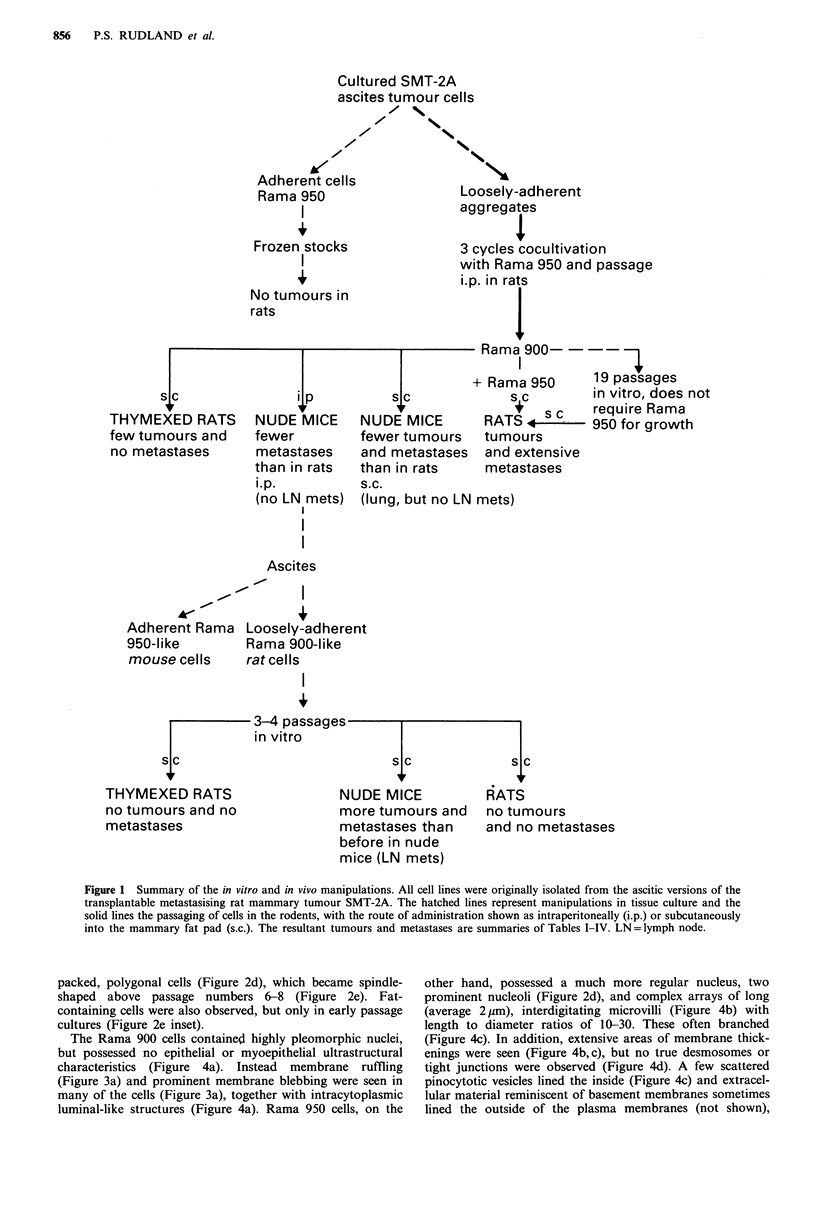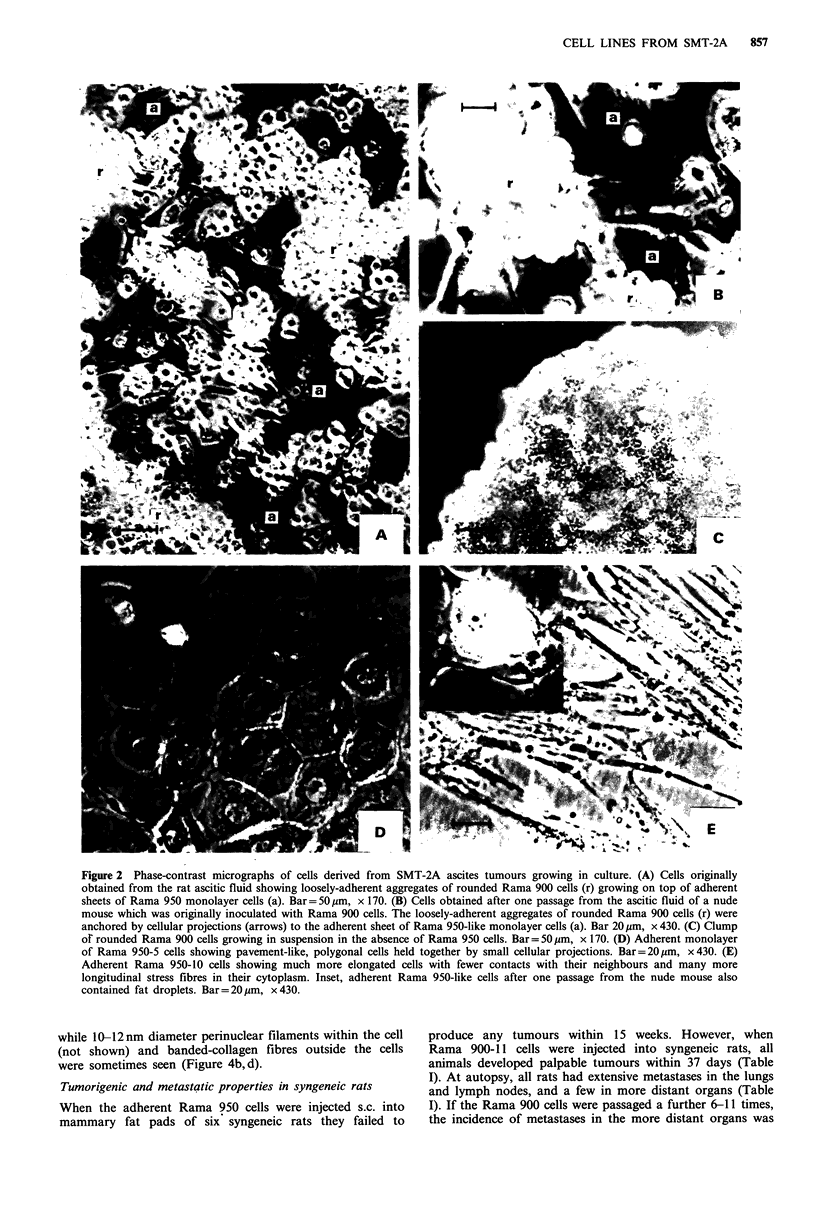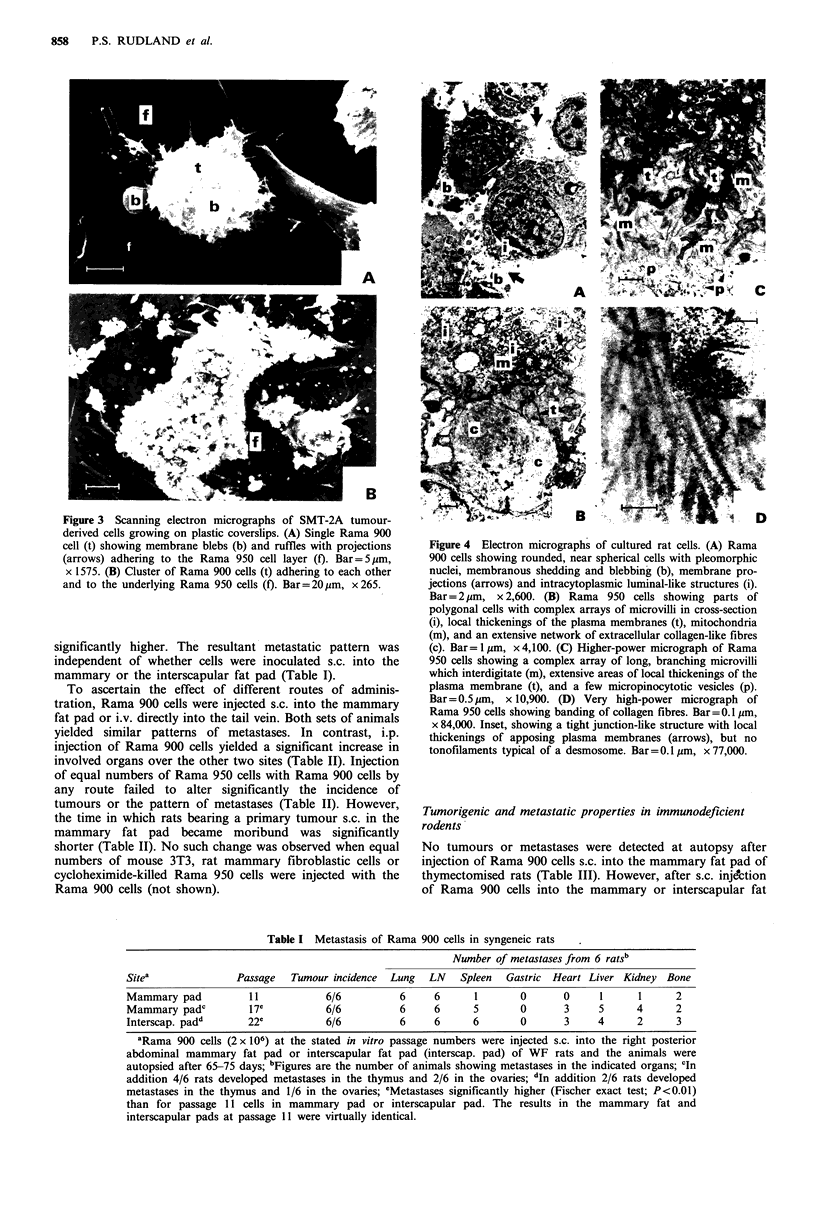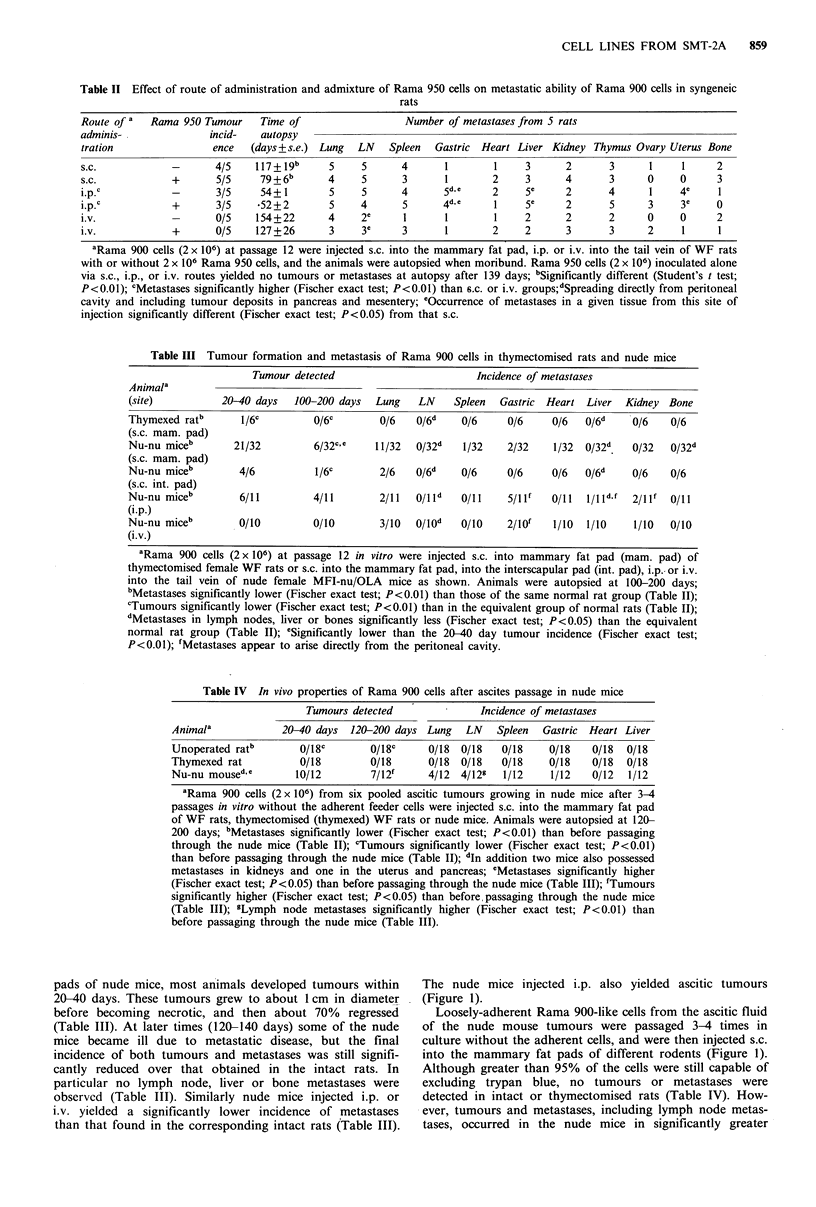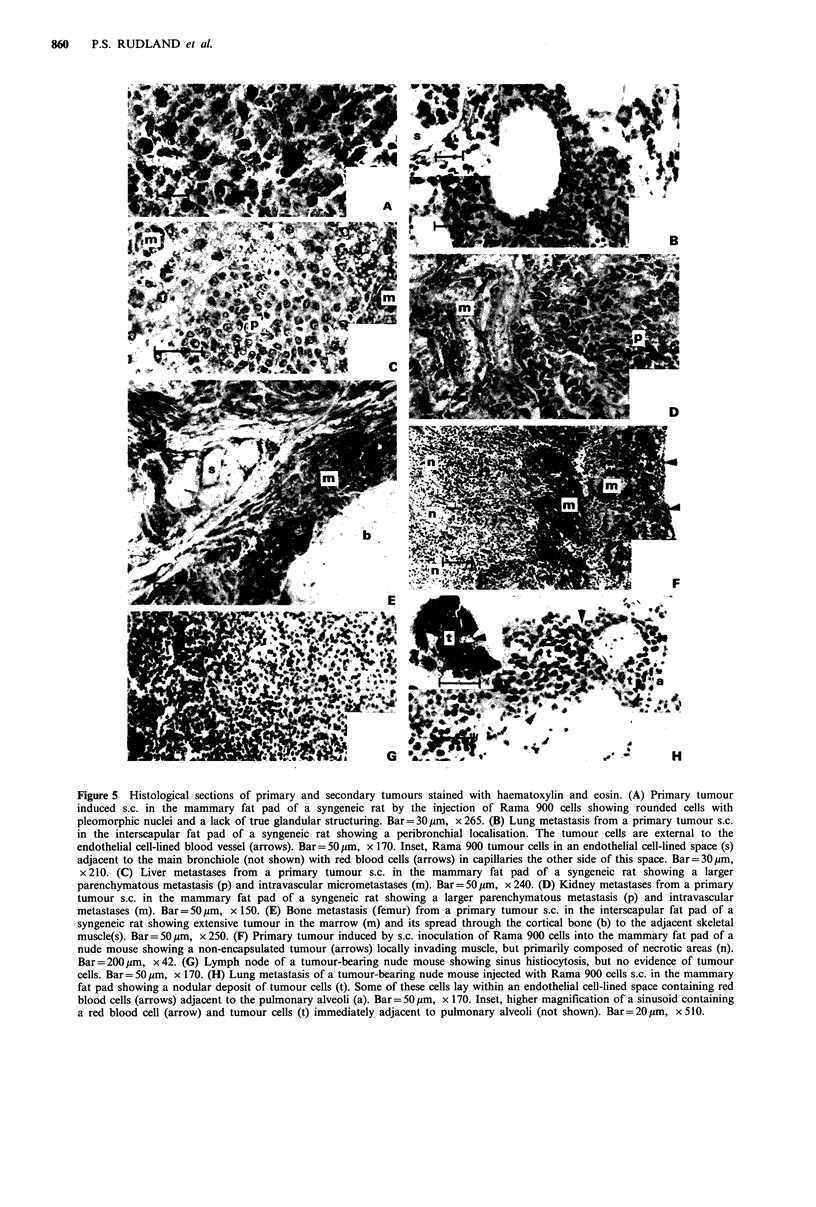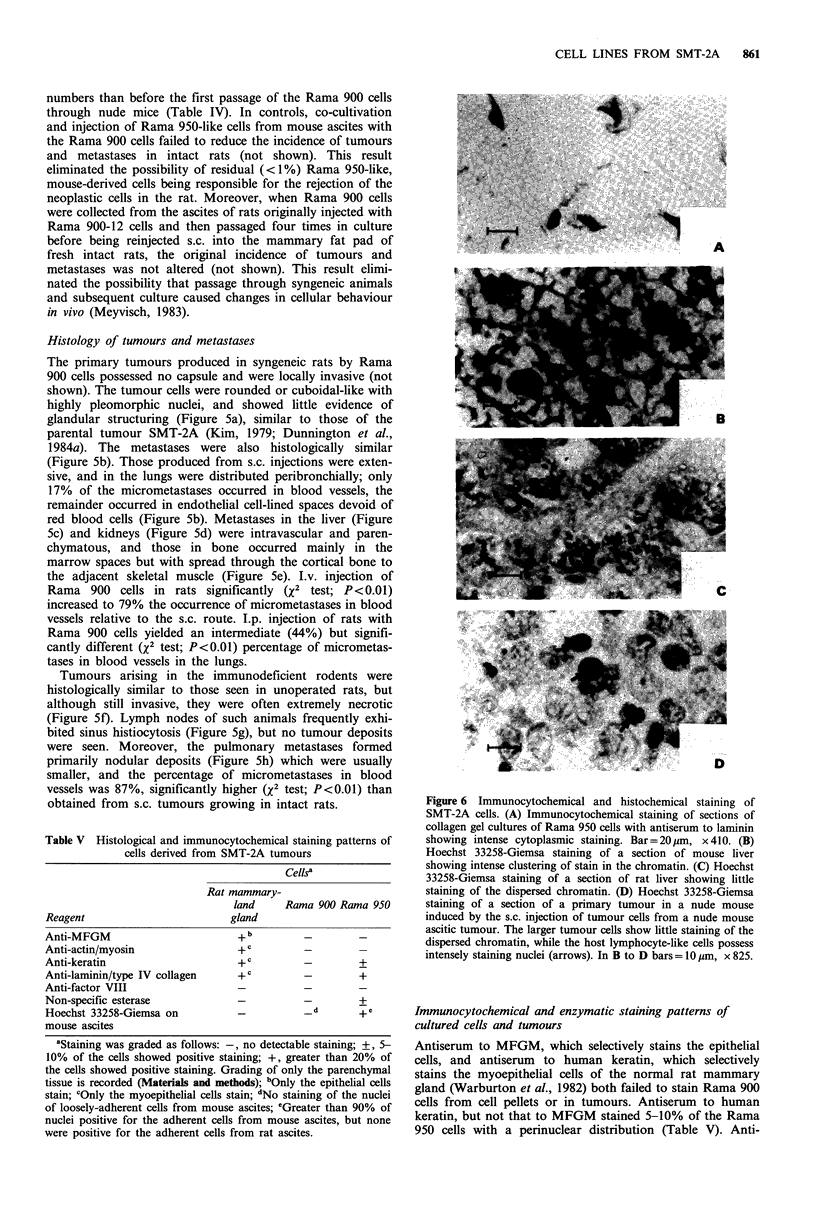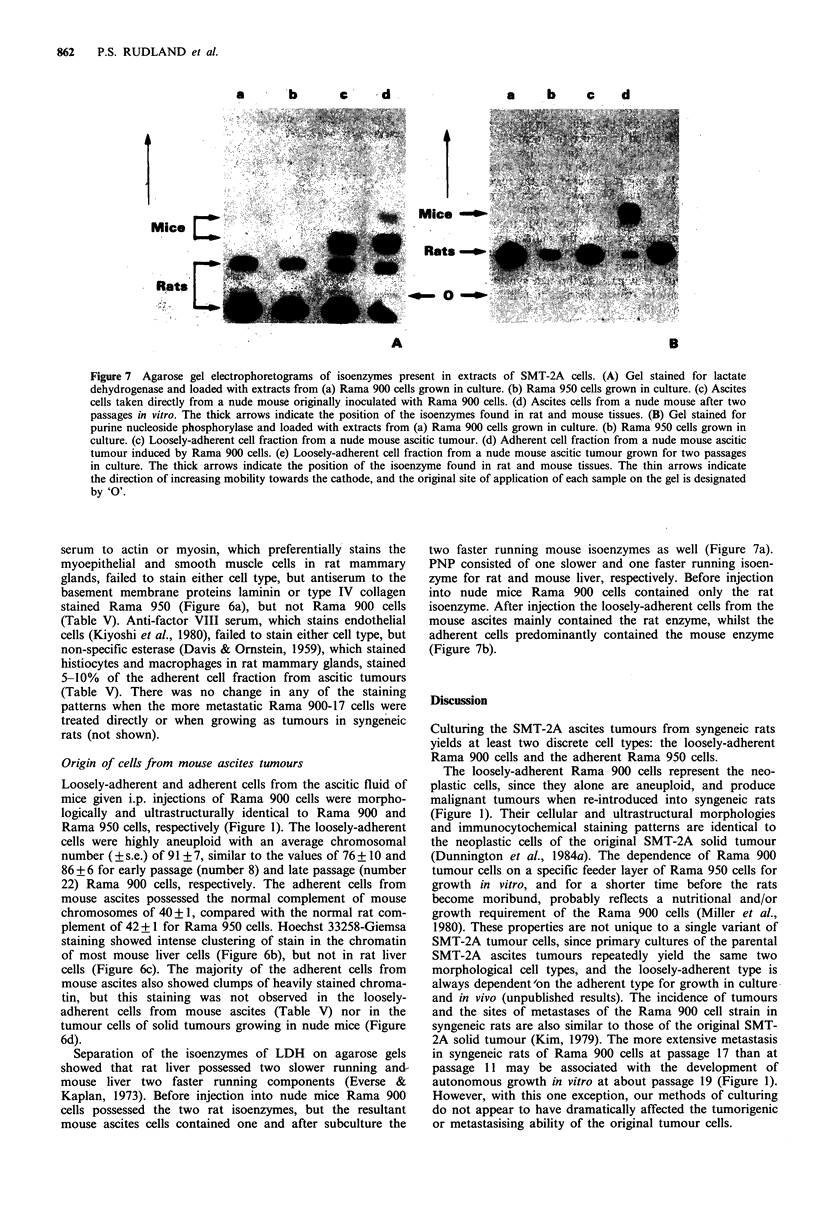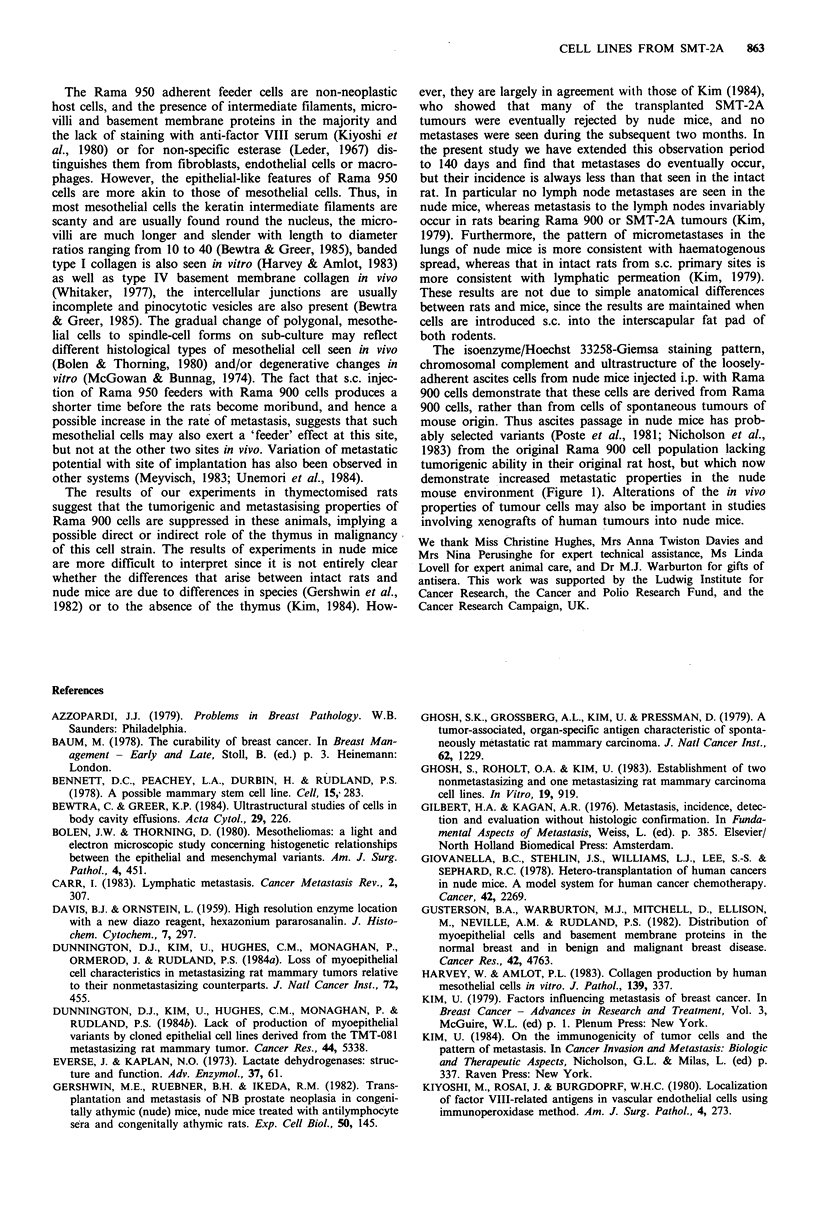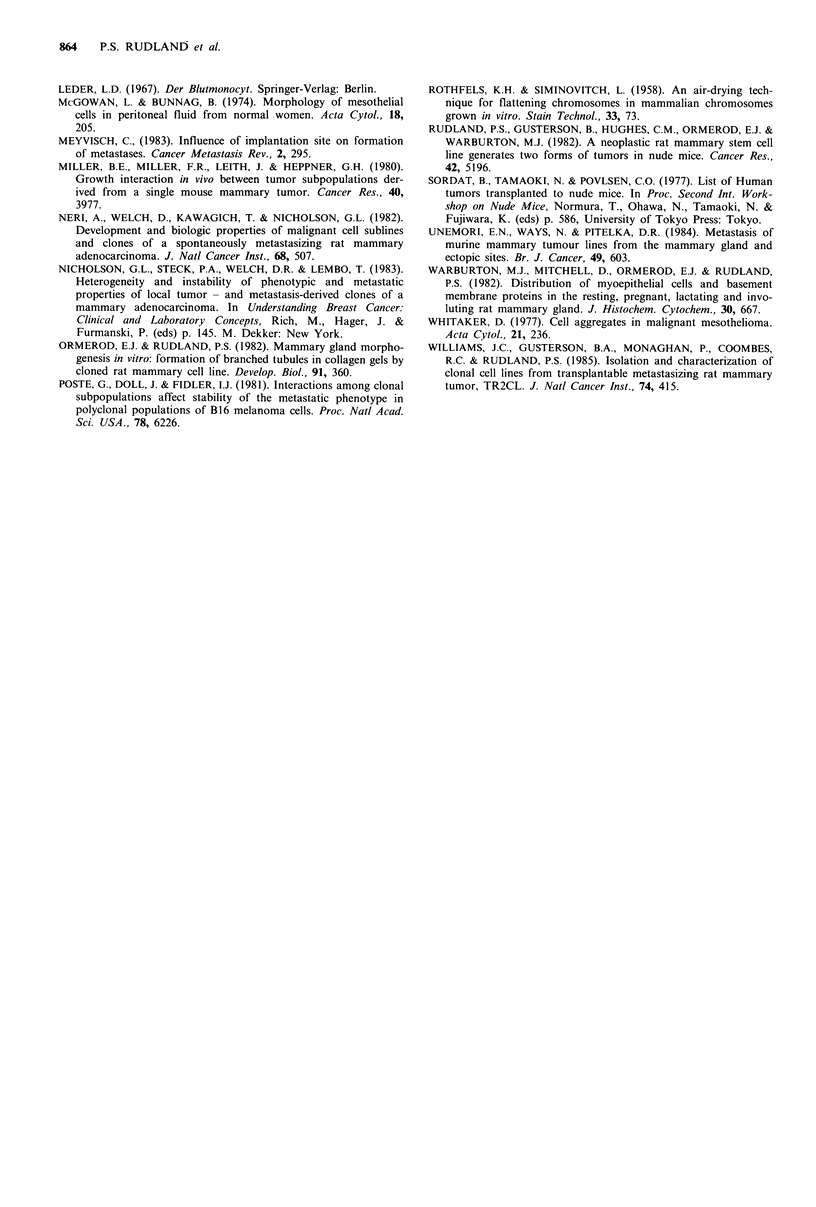# Isolation and properties of cell lines from the metastasising rat mammary tumour SMT-2A.

**DOI:** 10.1038/bjc.1989.182

**Published:** 1989-06

**Authors:** P. S. Rudland, D. J. Dunnington, U. Kim, B. A. Gusterson, M. J. O'Hare, P. Monaghan

**Affiliations:** Department of Biochemistry, Liverpool University, UK.

## Abstract

**Images:**


*Br. J. Cancer* (1989), **59**, 854–864 © The Macmillan Press Ltd., 1989

### Isolation and properties of cell lines from the metastasising rat mammary tumour SMT-2A

P.S. Rudland[1], D.J. Dunnington[2], U. Kim[3], B.A. Gusterson[4], M.J. O'Hare[4] & P. Monaghan[4]

[1]*Cancer and Polio Research Fund Laboratories, Department of Biochemistry, Liverpool University, Liverpool L69 3BX, UK;* [2]*Smith, Kline and French Laboratories, 1500 Spring Garden Street, Philadelphia, PA 19101, USA;* [3]*Roswell Park Memorial Institute, Buffalo, NY 14263, USA; and* [4]*Department of Pathology, Institute of Cancer Research, Sutton, Surrey SM2 5PX, UK.*

**Summary** A new cell line Rat mammary (Rama) 900 was isolated from the ascitic version of the SMT-2A metastasising rat mammary tumour by stepwise adaptation of the tumour cells to tissue culture. The cells grew mainly as loosely-adherent aggregates, and were dependent during the first 18 passages *in vitro* on a feeder layer of mesothelial-like cells (Rama 950) obtained from the same tumour. Subcutaneous injection of Rama 900 cells in fat pads of syngeneic Wistar Furth rats yielded anaplastic primary tumours and extensive, gross metastases including those in lungs, lymph nodes, liver and bones, similar to the parental transplantable tumour. The extent of metastatic spread from subcutaneous fat pads was increased by passage 17 *in vitro* for the Rama 900 cells. A similar extent of metastatic spread was achieved at earlier times by injecting the original cells with the non-tumorigenic Rama 950 cells *in vivo*. Subcutaneous injection of Rama 900 into thymectomised rats or MF1 nu/nu mice yielded fewer tumours, most of which regressed. No metastases occurred in the thymectomised rats and fewer metastases, mainly in lungs but not in lymph nodes, livers or bones, were seen in the nude mice. The ascitic tumours formed by intraperitoneal injection of nude mice contained both anaplastic rat cells similar to Rama 900 and mouse mesothelial-like cells similar to Rama 950. Although these anaplastic ascites cells failed to yield any tumours in syngeneic or thymectomised rats, they still produced tumours and metastases, including those in lymph nodes, in nude mice.

The clinical feature of breast cancer that makes successful treatment difficult is the high incidence of early metastases (Baum, 1978). Unfortunately measurement of the metastatic properties of cells derived from human breast cancers has been severely hampered by the lack of a suitable bioassay (Sordat *et al.*, 1977; Giovanella *et al.*, 1978). Thus model systems have been established in rodents for the bioassay of metastasising mammary cells in syngeneic animals. However, although several epithelial-like cell lines have been isolated from spontaneous (Neri *et al.*, 1982) and transplantable (Ghosh *et al.*, 1983; Dunnington *et al.*, 1984*b*; Williams *et al.*, 1985) metastasising rat mammary carcinomas, they produce, at best, gross metastases in only the lungs and lymph nodes from primary tumours growing in subcutaneous sites, with much of the dissemination occurring via the haematogenous route. In contrast human breast cancers usually disseminate widely throughout the body involving, in decreasing order of preference, lymph nodes, bone, lung, liver and brain (Gilbert & Kagan, 1976), and a major route of spread, at least in the early phases of the disease, is thought to be via the lymphatic system (Carr, 1983). Thus, most of the available animal model systems for breast cancer largely fail to disseminate like the human disease.

One rare example of a transplantable rat mammary tumour that does show widespread metastatic properties is SMT-2A (Kim, 1979). This transplantable tumour disseminates predominantly via the lymphatic system in Wistar Furth (WF) rats giving rise to widespread, gross metastases, including those in liver and bones, as well as those in lungs and lymph nodes (Kim, 1979). Moreover, its tumours and metastases are composed of anaplastic cells of an epithelial (Ghosh *et al.*, 1979), and not a myoepithelial origin (Dunnington *et al.*, 1984*a*), like malignant breast disease in humans (Azzopardi, 1979; Gusterson *et al.*, 1982). This system therefore simulates to a large degree the pattern of metastatic spread and the cellular pathology often encountered in human ductal carcinomas. The SMT-2A tumours also contain extensive numbers of non-neoplastic cells (Kim, 1979; Dunnington *et al.*, 1984*a*). We now investigate the different viable cell types present in the SMT-2A tumour by isolating them as cell lines in tissue culture and characterising them with respect to morphological criteria and to their tumorigenic and metastatic properties when introduced into syngeneic rats and immune-deprived rodents.

### Materials and methods

### *Animals and transplantable tumour*

Female Ludwig-Wistar (WF) rats were obtained from Olac Ltd, Banbury, Oxfordshire, UK, and maintained as before (Dunnington *et al.*, 1984*b*). Female nude mice of the MF1-nu/OLA strain were obtained from the same supplier and were specifically pathogen-free. They were maintained on Labsure CRM Irradiated Diet (RMH Agriculture Ltd, Poole, Dorset, UK) and sterile (autoclaved) tap water. Female WF rats were also thymectomised under anaesthesia and were allowed 1 week for recovery before experimentation. These mice and rats were confirmed to be immunodeficient by their ability to grow tumours when injected with the allogeneic Rama 25 cells from Sprague Dawley rats (Bennett *et al.*, 1978); normal MF1/OLA mice and WF rats failed to do so.

The SMT-2A metastasising rat mammary tumour is a transplantable tumour line which is syngeneic to the WF rat strain and is carried either as solid or ascitic form (Kim, 1979). When injected s.c. into the mammary fat pads of syngeneic rats, both forms produce solid tumours. The tumours metastasise primarily via the lymphatic route to the lymph nodes, lungs, kidneys, liver, spleen and bone (Kim, 1979). The ascitic form of the tumour was used because a suspension of single cells can readily be obtained from ascites fluid without recourse to the damaging digestion procedures necessary to prepare cells from solid tumours.

Correspondence: P.S. Rudland.
Animals were maintained under the guidelines set forth by the Ludwig Institute for Cancer Research, Sutton, Surrey, UK.

*Preparation of cell strains from the SMT-2A tumour*

Rats bearing SMT-2A ascites tumours were anaesthetised and the ascites fluid was removed with a sterile syringe. The fluid was centrifuged at 200*g* for 1.5 min through 20 ml of phosphate-buffered saline ($Ca^{2+}$ and $Mg^{2+}$-free) (PBS). The pellet was suspended in routine medium (RM = Dulbecco's modified Eagles Medium (DEM), 5% fetal calf serum and 50 ng $ml^{-1}$ each of hydrocortisone and insulin), and plated at approximately $10^4$ cells per 5 cm diameter tissue culture dish. After 24 h in culture, loosely-adherent cells were removed by pipetting with fresh medium, and the cultures of adherent cells were allowed to grow to confluence. The resulting cell strain, termed Rama 950 (Figure 1), was grown in RM, passaged 1:4 by treatment with EDTA/trypsin solutions (Bennett *et al.*, 1978) and stored frozen in liquid nitrogen.

The loosely-adherent cells from above were cultured for 24 h in RM with Rama 950 and injected i.p. into rats. After 4 weeks, cells from the resulting tumours were cultured for 4 days in RM, before they were again re-introduced into animals. After further growth as tumours for another 4 weeks, the cells were cultured for 3 weeks in RM, injected into animals, and cells from the resulting tumours were frozen and stored in liquid nitrogen. The Rama 900 cell line was obtained by thawing and culturing a sample of cells from this third selection cycle on Rama 950 as above (Figure 1). The RM was changed every 3–4 days until large clusters were formed, and the cultures were routinely passaged 1:3 by a brief, 1 min treatment with EDTA/trypsin solutions. The original medium, the PBS-washings, and the detached cells were pooled and cells were collected by centrifugation at 200*g* for 5 min before each transfer. Morphological appearance of living cultures was observed and photographed as before (Dunnington *et al.*, 1984*b*).

*Tumorigenic and metastatic studies*

Cultured Rama 900 cells for injection into 6–9-week-old syngeneic or thymectomised rats or 8–10-week-old nude mice were separated from the Rama 950 cells by vigorous pipetting with RM. Cells were washed by centrifugation through PBS and $2 \times 10^6$ were injected into each animal via one of the following routes: s.c. into the region of the right posterior abdominal mammary gland, s.c. into the interscapular fat pad, i.p. or i.v. into the tail vein (Dunnington *et al.*, 1984*b*). Tumour-bearing animals were killed when moribund and the lungs, lymph nodes (axillary and paraaortic), spleen, liver, kidneys including adrenals, thymus, heart, uterus, ovaries, stomach plus colon (gastric), pancreas and skeleton (femur, sternum, spine) were examined for gross metastases.

Samples of the above organs and any other abnormal tissues were fixed in Methacarn and processed for histology (Warburton *et al.*, 1982). Non-tumour-bearing animals were autopsied after a period of at least 15 weeks from the time of injection, and tissue from the site of injection was also fixed and processed as above. The percentage of micrometastases ($<1\,mm^2$) in blood vessels in the lungs was estimated from two longitudinal histological sections, the criterion of a blood vessel being the presence of red blood cells in a vascular space.

*Immunocytochemical and enzymatic staining*

For immunocytochemical staining, two separate cell pellets each of Rama 900 passaged in culture 12 times (Rama 900-12) or Rama 950-4 were prepared either by pipetting off cells or by releasing them with EDTA respectively, and then centrifuging them through PBS. Five primary tumours were produced by injection of Rama 900-12 cells into rat mammary fat pads, and Rama 950-4 cells were grown on two 0.3% (w/v) collagen gels (Ormerod & Rudland, 1982). All specimens were fixed in Methacarn and sectioned, and the sections were incubated with antisera to rat milk fat globule membrane (MFGM), actin, myosin, human callus keratin, laminin, type IV collagen, factor VIII (Kiyoshi *et al.*, 1980), and processed as before (Warburton *et al.*, 1982). Pellets of cells were also frozen and cryostat sections were stained for non-specific esterase (Davis & Ornstein, 1959). Five fields from two sections of each specimen for each reagent were examined by two independent observers, and the average result of the percentage of a given cell type that was stained was recorded and photographed (Dunnington *et al.*, 1984*a*, *b*). The specificity of staining was checked by the same controls as before (Warburton *et al.*, 1982).

For analysis of cells in ascites tumours, cultured cells or separated cell-fractions of loosely-adherent and EDTA-released adherent cells from mouse and rat ascites fluids were collected by centrifugation through PBS. Cell pellets, tumour samples and control fragments of rat and mouse livers were either fixed in 10% (v/v) formaldehyde in PBS, sectioned and processed for Hoechst 33258-Giemsa staining (Rudland *et al.*, 1982), or lysed with neutral detergents and the soluble extracts electrophoresed on prepacked horizontal agarose gels at pH 8.6 (AuthentiKit System, Corning, USA). The gels were subsequently stained for lactic dehydrogenase (LDH) (EC 1.1.1.27) or for purine nucleotide phosphorylase (PNP) (EC 2.4.2.1) exactly as described in the AuthentiKit manual. Photographs of the agarose gels were recorded with a Polaroid quarter plate camera on type 55 film.

Chromosomal spreads were prepared according to Rothfels & Siminovitch (1958) and a minimum of 100 were counted.

*Electron microscopy*

For scanning electron microscopy, Rama 900 tumour cells were plated on to Rama 950 cells growing on plastic coverslips (Lux Scientific Corp., USA) and cultured for 3 days in RM before being fixed in 2% (w/v) glutaraldehyde. Cells were then processed for scanning electron microscopy (Dunnington *et al.*, 1984*b*).

For transmission electron microscopy, Rama 900-12 cells were released from the adherent Rama 950 cells by pipetting, and Rama 950-3 were cultured in the absence of Rama 900 and removed from the substratum by scraping with a rubber spatula. In addition, the loosely-adherent and the EDTA-released adherent cell fraction from rat and mouse ascitic fluids were also collected. All cells were washed by centrifugation through PBS, and then fixed with 2% (w/v) glutaraldehyde in phosphate buffer before being processed for transmission electron microscopy (Ormerod & Rudland, 1982).

### Results

*Morphology of cultured cells*

When grown in culture SMT-2A ascites cells yielded loosely-adherent aggregates of rounded cells and adherent sheets of monolayer cells (Figures 2a and 3a). The rounded cells produced 5–10 times as many filopodia to the monolayer (Figure 3a) and to each other (not shown) as to the substratum, resulting in the formation of large clusters (Figures 2b and 3b). Sometimes the rounded cells detached completely from the monolayer cells, forming free-floating single cells and clumps of rounded cells (Figure 2c). When these non-adherent cells and clumps were replated on to the established monolayers, they continued to proliferate, yielding both the loosely-adherent aggregates and the free-floating forms. The loosely-adherent/floating cells were absolutely dependent on the monolayer cells for continued survival during the first 18 passages *in vitro*. The former were termed Rama 900 and the latter Rama 950 cells (Figure 1). The Rama 950 cells could not be replaced by mouse 3T3 or rat fibroblastic cell lines for growth of Rama 900 cells (not shown). However, at passage 19 the Rama 900 cell clumps could be grown in the absence of Rama 950 cells (Figure 2c). The adherent Rama 950 cells consisted initially of close-

**Figure 1** Summary of the *in vitro* and *in vivo* manipulations. All cell lines were originally isolated from the ascitic versions of the transplantable metastasising rat mammary tumour SMT-2A. The hatched lines represent manipulations in tissue culture and the solid lines the passaging of cells in the rodents, with the route of administration shown as intraperitoneally (i.p.) or subcutaneously into the mammary fat pad (s.c.). The resultant tumours and metastases are summaries of Tables I–IV. LN = lymph node.

packed, polygonal cells (Figure 2d), which became spindle-shaped above passage numbers 6–8 (Figure 2e). Fat-containing cells were also observed, but only in early passage cultures (Figure 2e inset).

The Rama 900 cells contained highly pleomorphic nuclei, but possessed no epithelial or myoepithelial ultrastructural characteristics (Figure 4a). Instead membrane ruffling (Figure 3a) and prominent membrane blebbing were seen in many of the cells (Figure 3a), together with intracytoplasmic luminal-like structures (Figure 4a). Rama 950 cells, on the other hand, possessed a much more regular nucleus, two prominent nucleoli (Figure 2d), and complex arrays of long (average 2 $\mu$m), interdigitating microvilli (Figure 4b) with length to diameter ratios of 10–30. These often branched (Figure 4c). In addition, extensive areas of membrane thickenings were seen (Figure 4b, c), but no true desmosomes or tight junctions were observed (Figure 4d). A few scattered pinocytotic vesicles lined the inside (Figure 4c) and extracellular material reminiscent of basement membranes sometimes lined the outside of the plasma membranes (not shown),

**Figure 2** Phase-contrast micrographs of cells derived from SMT-2A ascites tumours growing in culture. (**A**) Cells originally obtained from the rat ascitic fluid showing loosely-adherent aggregates of rounded Rama 900 cells (r) growing on top of adherent sheets of Rama 950 monolayer cells (a). Bar = 50 μm, × 170. (**B**) Cells obtained after one passage from the ascitic fluid of a nude mouse which was originally inoculated with Rama 900 cells. The loosely-adherent aggregates of rounded Rama 900 cells (r) were anchored by cellular projections (arrows) to the adherent sheet of Rama 950-like monolayer cells (a). Bar 20 μm, × 430. (**C**) Clump of rounded Rama 900 cells growing in suspension in the absence of Rama 950 cells. Bar = 50 μm, × 170. (**D**) Adherent monolayer of Rama 950-5 cells showing pavement-like, polygonal cells held together by small cellular projections. Bar = 20 μm, × 430. (**E**) Adherent Rama 950-10 cells showing much more elongated cells with fewer contacts with their neighbours and many more longitudinal stress fibres in their cytoplasm. Inset, adherent Rama 950-like cells after one passage from the nude mouse also contained fat droplets. Bar = 20 μm, × 430.

while 10–12 nm diameter perinuclear filaments within the cell (not shown) and banded-collagen fibres outside the cells were sometimes seen (Figure 4b, d).

*Tumorigenic and metastatic properties in syngeneic rats*

When the adherent Rama 950 cells were injected s.c. into mammary fat pads of six syngeneic rats they failed to produce any tumours within 15 weeks. However, when Rama 900-11 cells were injected into syngeneic rats, all animals developed palpable tumours within 37 days (Table I). At autopsy, all rats had extensive metastases in the lungs and lymph nodes, and a few in more distant organs (Table I). If the Rama 900 cells were passaged a further 6–11 times, the incidence of metastases in the more distant organs was

**Figure 3** Scanning electron micrographs of SMT-2A tumour-derived cells growing on plastic coverslips. (**A**) Single Rama 900 cell (t) showing membrane blebs (b) and ruffles with projections (arrows) adhering to the Rama 950 cell layer (f). Bar = 5 $\mu$m, × 1575. (**B**) Cluster of Rama 900 cells (t) adhering to each other and to the underlying Rama 950 cells (f). Bar = 20 $\mu$m, × 265.

**Figure 4** Electron micrographs of cultured rat cells. (**A**) Rama 900 cells showing rounded, near spherical cells with pleomorphic nuclei, membranous shedding and blebbing (b), membrane projections (arrows) and intracytoplasmic luminal-like structures (i). Bar = 2 $\mu$m, × 2,600. (**B**) Rama 950 cells showing parts of polygonal cells with complex arrays of microvilli in cross-section (i), local thickenings of the plasma membranes (t), mitochondria (m), and an extensive network of extracellular collagen-like fibres (c). Bar = 1 $\mu$m, × 4,100. (**C**) Higher-power micrograph of Rama 950 cells showing a complex array of long, branching microvilli which interdigitate (m), extensive areas of local thickenings of the plasma membrane (t), and a few micropinocytotic vesicles (p). Bar = 0.5 $\mu$m, × 10,900. (**D**) Very high-power micrograph of Rama 950 cells showing banding of collagen fibres. Bar = 0.1 $\mu$m, × 84,000. Inset, showing a tight junction-like structure with local thickenings of apposing plasma membranes (arrows), but no tonofilaments typical of a desmosome. Bar = 0.1 $\mu$m, × 77,000.

significantly higher. The resultant metastatic pattern was independent of whether cells were inoculated s.c. into the mammary or the interscapular fat pad (Table I).

To ascertain the effect of different routes of administration, Rama 900 cells were injected s.c. into the mammary fat pad or i.v. directly into the tail vein. Both sets of animals yielded similar patterns of metastases. In contrast, i.p. injection of Rama 900 cells yielded a significant increase in involved organs over the other two sites (Table II). Injection of equal numbers of Rama 950 cells with Rama 900 cells by any route failed to alter significantly the incidence of tumours or the pattern of metastases (Table II). However, the time in which rats bearing a primary tumour s.c. in the mammary fat pad became moribund was significantly shorter (Table II). No such change was observed when equal numbers of mouse 3T3, rat mammary fibroblastic cells or cycloheximide-killed Rama 950 cells were injected with the Rama 900 cells (not shown).

*Tumorigenic and metastatic properties in immunodeficient rodents*

No tumours or metastases were detected at autopsy after injection of Rama 900 cells s.c. into the mammary fat pad of thymectomised rats (Table III). However, after s.c. injection of Rama 900 cells into the mammary or interscapular fat

**Table I** Metastasis of Rama 900 cells in syngeneic rats

| | | | *Number of metastases from 6 rats*[b] | | | | | | | |
|---|---|---|---|---|---|---|---|---|---|---|
| *Site*[a] | *Passage* | *Tumour incidence* | *Lung* | *LN* | *Spleen* | *Gastric* | *Heart* | *Liver* | *Kidney* | *Bone* |
| Mammary pad | 11 | 6/6 | 6 | 6 | 1 | 0 | 0 | 1 | 1 | 2 |
| Mammary pad[c] | 17[e] | 6/6 | 6 | 6 | 5 | 0 | 3 | 5 | 4 | 2 |
| Interscap. pad[d] | 22[e] | 6/6 | 6 | 6 | 6 | 0 | 3 | 4 | 2 | 3 |

[a]Rama 900 cells ($2 \times 10^6$) at the stated *in vitro* passage numbers were injected s.c. into the right posterior abdominal mammary fat pad or interscapular fat pad (interscap. pad) of WF rats and the animals were autopsied after 65–75 days; [b]Figures are the number of animals showing metastases in the indicated organs; [c]In addition 4/6 rats developed metastases in the thymus and 2/6 in the ovaries; [d]In addition 2/6 rats developed metastases in the thymus and 1/6 in the ovaries; [e]Metastases significantly higher (Fischer exact test; $P < 0.01$) than for passage 11 cells in mammary pad or interscapular pad. The results in the mammary fat and interscapular pads at passage 11 were virtually identical.

**Table II** Effect of route of administration and admixture of Rama 950 cells on metastatic ability of Rama 900 cells in syngeneic rats

| *Route of*[a] *adminis-tration* | *Rama 950* | *Tumour incid-ence* | *Time of autopsy (days ± s.e.)* | *Number of metastases from 5 rats* | | | | | | | | | | |
|---|---|---|---|---|---|---|---|---|---|---|---|---|---|---|
| | | | | *Lung* | *LN* | *Spleen* | *Gastric* | *Heart* | *Liver* | *Kidney* | *Thymus* | *Ovary* | *Uterus* | *Bone* |
| s.c. | – | 4/5 | 117±19[b] | 5 | 5 | 4 | 1 | 1 | 3 | 2 | 3 | 1 | 1 | 2 |
| s.c. | + | 5/5 | 79±6[b] | 4 | 5 | 3 | 1 | 2 | 3 | 4 | 3 | 0 | 0 | 3 |
| i.p.[c] | – | 3/5 | 54±1 | 5 | 5 | 4 | 5[d,e] | 2 | 5[e] | 2 | 4 | 1 | 4[e] | 1 |
| i.p.[c] | + | 3/5 | ·52±2 | 5 | 4 | 5 | 4[d,e] | 1 | 5[e] | 2 | 5 | 3 | 3[e] | 0 |
| i.v. | – | 0/5 | 154±22 | 4 | 2[e] | 1 | 1 | 1 | 2 | 2 | 2 | 0 | 0 | 2 |
| i.v. | + | 0/5 | 127±26 | 3 | 3[e] | 3 | 1 | 2 | 2 | 3 | 3 | 2 | 1 | 1 |

[a]Rama 900 cells ($2 \times 10^6$) at passage 12 were injected s.c. into the mammary fat pad, i.p. or i.v. into the tail vein of WF rats with or without $2 \times 10^6$ Rama 950 cells, and the animals were autopsied when moribund. Rama 950 cells ($2 \times 10^6$) inoculated alone via s.c., i.p., or i.v. routes yielded no tumours or metastases at autopsy after 139 days; [b]Significantly different (Student's *t* test; $P<0.01$); [c]Metastases significantly higher (Fischer exact test; $P<0.01$) than s.c. or i.v. groups;[d]Spreading directly from peritoneal cavity and including tumour deposits in pancreas and mesentery; [e]Occurrence of metastases in a given tissue from this site of injection significantly different (Fischer exact test; $P<0.05$) from that s.c.

**Table III** Tumour formation and metastasis of Rama 900 cells in thymectomised rats and nude mice

| *Animal*[a] *(site)* | *Tumour detected* | | *Incidence of metastases* | | | | | | | |
|---|---|---|---|---|---|---|---|---|---|---|
| | *20–40 days* | *100–200 days* | *Lung* | *LN* | *Spleen* | *Gastric* | *Heart* | *Liver* | *Kidney* | *Bone* |
| Thymexed rat[b] (s.c. mam. pad) | 1/6[c] | 0/6[c] | 0/6 | 0/6[d] | 0/6 | 0/6 | 0/6 | 0/6[d] | 0/6 | 0/6 |
| Nu-nu mice[b] (s.c. mam. pad) | 21/32 | 6/32[c,e] | 11/32 | 0/32[d] | 1/32 | 2/32 | 1/32 | 0/32[d] | 0/32 | 0/32[d] |
| Nu-nu mice[b] (s.c. int. pad) | 4/6 | 1/6[c] | 2/6 | 0/6[d] | 0/6 | 0/6 | 0/6 | 0/6[d] | 0/6 | 0/6 |
| Nu-nu mice[b] (i.p.) | 6/11 | 4/11 | 2/11 | 0/11[d] | 0/11 | 5/11[f] | 0/11 | 1/11[d,f] | 2/11[f] | 0/11 |
| Nu-nu mice[b] (i.v.) | 0/10 | 0/10 | 3/10 | 0/10[d] | 0/10 | 2/10[f] | 1/10 | 1/10 | 1/10 | 0/10 |

[a]Rama 900 cells ($2 \times 10^6$) at passage 12 *in vitro* were injected s.c. into mammary fat pad (mam. pad) of thymectomised female WF rats or s.c. into the mammary fat pad, into the interscapular pad (int. pad), i.p. or i.v. into the tail vein of nude female MFI-nu/OLA mice as shown. Animals were autopsied at 100–200 days; [b]Metastases significantly lower (Fischer exact test; $P<0.01$) than those of the same normal rat group (Table II); [c]Tumours significantly lower (Fischer exact test; $P<0.01$) than in the equivalent group of normal rats (Table II); [d]Metastases in lymph nodes, liver or bones significantly less (Fischer exact test; $P<0.05$) than the equivalent normal rat group (Table II); [e]Significantly lower than the 20–40 day tumour incidence (Fischer exact test; $P<0.01$); [f]Metastases appear to arise directly from the peritoneal cavity.

**Table IV** *In vivo* properties of Rama 900 cells after ascites passage in nude mice

| *Animal*[a] | *Tumours detected* | | *Incidence of metastases* | | | | | |
|---|---|---|---|---|---|---|---|---|
| | *20–40 days* | *120–200 days* | *Lung* | *LN* | *Spleen* | *Gastric* | *Heart* | *Liver* |
| Unoperated rat[b] | 0/18[c] | 0/18[c] | 0/18 | 0/18 | 0/18 | 0/18 | 0/18 | 0/18 |
| Thymexed rat | 0/18 | 0/18 | 0/18 | 0/18 | 0/18 | 0/18 | 0/18 | 0/18 |
| Nu-nu mouse[d,e] | 10/12 | 7/12[f] | 4/12 | 4/12[g] | 1/12 | 1/12 | 0/12 | 1/12 |

[a]Rama 900 cells ($2 \times 10^6$) from six pooled ascitic tumours growing in nude mice after 3–4 passages *in vitro* without the adherent feeder cells were injected s.c. into the mammary fat pad of WF rats, thymectomised (thymexed) WF rats or nude mice. Animals were autopsied at 120–200 days; [b]Metastases significantly lower (Fischer exact test; $P<0.01$) than before passaging through the nude mice (Table II); [c]Tumours significantly lower (Fischer exact test; $P<0.01$) than before passaging through the nude mice (Table II); [d]In addition two mice also possessed metastases in kidneys and one in the uterus and pancreas; [e]Metastases significantly higher (Fischer exact test; $P<0.05$) than before passaging through the nude mice (Table III); [f]Tumours significantly higher (Fischer exact test; $P<0.05$) than before passaging through the nude mice (Table III); [g]Lymph node metastases significantly higher (Fischer exact test; $P<0.01$) than before passaging through the nude mice (Table III).

pads of nude mice, most animals developed tumours within 20–40 days. These tumours grew to about 1 cm in diameter before becoming necrotic, and then about 70% regressed (Table III). At later times (120–140 days) some of the nude mice became ill due to metastatic disease, but the final incidence of both tumours and metastases was still significantly reduced over that obtained in the intact rats. In particular no lymph node, liver or bone metastases were observed (Table III). Similarly nude mice injected i.p. or i.v. yielded a significantly lower incidence of metastases than that found in the corresponding intact rats (Table III). The nude mice injected i.p. also yielded ascitic tumours (Figure 1).

Loosely-adherent Rama 900-like cells from the ascitic fluid of the nude mouse tumours were passaged 3–4 times in culture without the adherent cells, and were then injected s.c. into the mammary fat pads of different rodents (Figure 1). Although greater than 95% of the cells were still capable of excluding trypan blue, no tumours or metastases were detected in intact or thymectomised rats (Table IV). However, tumours and metastases, including lymph node metastases, occurred in the nude mice in significantly greater

**Figure 5** Histological sections of primary and secondary tumours stained with haematoxylin and eosin. (**A**) Primary tumour induced s.c. in the mammary fat pad of a syngeneic rat by the injection of Rama 900 cells showing rounded cells with pleomorphic nuclei and a lack of true glandular structuring. Bar = 30 $\mu$m, ×265. (**B**) Lung metastasis from a primary tumour s.c. in the interscapular fat pad of a syngeneic rat showing a peribronchial localisation. The tumour cells are external to the endothelial cell-lined blood vessel (arrows). Bar = 50 $\mu$m, ×170. Inset, Rama 900 tumour cells in an endothelial cell-lined space (s) adjacent to the main bronchiole (not shown) with red blood cells (arrows) in capillaries the other side of this space. Bar = 30 $\mu$m, ×210. (**C**) Liver metastases from a primary tumour s.c. in the mammary fat pad of a syngeneic rat showing a larger parenchymatous metastasis (p) and intravascular micrometastases (m). Bar = 50 $\mu$m, ×240. (**D**) Kidney metastases from a primary tumour s.c. in the mammary fat pad of a syngeneic rat showing a larger parenchymatous metastasis (p) and intravascular metastases (m). Bar = 50 $\mu$m, ×150. (**E**) Bone metastasis (femur) from a primary tumour s.c. in the interscapular fat pad of a syngeneic rat showing extensive tumour in the marrow (m) and its spread through the cortical bone (b) to the adjacent skeletal muscle(s). Bar = 50 $\mu$m, ×250. (**F**) Primary tumour induced by s.c. inoculation of Rama 900 cells into the mammary fat pad of a nude mouse showing a non-encapsulated tumour (arrows) locally invading muscle, but primarily composed of necrotic areas (n). Bar = 200 $\mu$m, ×42. (**G**) Lymph node of a tumour-bearing nude mouse showing sinus histiocytosis, but no evidence of tumour cells. Bar = 50 $\mu$m, ×170. (**H**) Lung metastasis of a tumour-bearing nude mouse injected with Rama 900 cells s.c. in the mammary fat pad showing a nodular deposit of tumour cells (t). Some of these cells lay within an endothelial cell-lined space containing red blood cells (arrows) adjacent to the pulmonary alveoli (a). Bar = 50 $\mu$m, ×170. Inset, higher magnification of a sinusoid containing a red blood cell (arrow) and tumour cells (t) immediately adjacent to pulmonary alveoli (not shown). Bar = 20 $\mu$m, ×510.

numbers than before the first passage of the Rama 900 cells through nude mice (Table IV). In controls, co-cultivation and injection of Rama 950-like cells from mouse ascites with the Rama 900 cells failed to reduce the incidence of tumours and metastases in intact rats (not shown). This result eliminated the possibility of residual (<1%) Rama 950-like, mouse-derived cells being responsible for the rejection of the neoplastic cells in the rat. Moreover, when Rama 900 cells were collected from the ascites of rats originally injected with Rama 900-12 cells and then passaged four times in culture before being reinjected s.c. into the mammary fat pad of fresh intact rats, the original incidence of tumours and metastases was not altered (not shown). This result eliminated the possibility that passage through syngeneic animals and subsequent culture caused changes in cellular behaviour *in vivo* (Meyvisch, 1983).

### *Histology of tumours and metastases*

The primary tumours produced in syngeneic rats by Rama 900 cells possessed no capsule and were locally invasive (not shown). The tumour cells were rounded or cuboidal-like with highly pleomorphic nuclei, and showed little evidence of glandular structuring (Figure 5a), similar to those of the parental tumour SMT-2A (Kim, 1979; Dunnington *et al.*, 1984*a*). The metastases were also histologically similar (Figure 5b). Those produced from s.c. injections were extensive, and in the lungs were distributed peribronchially; only 17% of the micrometastases occurred in blood vessels, the remainder occurred in endothelial cell-lined spaces devoid of red blood cells (Figure 5b). Metastases in the liver (Figure 5c) and kidneys (Figure 5d) were intravascular and parenchymatous, and those in bone occurred mainly in the marrow spaces but with spread through the cortical bone to the adjacent skeletal muscle (Figure 5e). I.v. injection of Rama 900 cells in rats significantly ($\chi^2$ test; $P<0.01$) increased to 79% the occurrence of micrometastases in blood vessels relative to the s.c. route. I.p. injection of rats with Rama 900 cells yielded an intermediate (44%) but significantly different ($\chi^2$ test; $P<0.01$) percentage of micrometastases in blood vessels in the lungs.

Tumours arising in the immunodeficient rodents were histologically similar to those seen in unoperated rats, but although still invasive, they were often extremely necrotic (Figure 5f). Lymph nodes of such animals frequently exhibited sinus histiocytosis (Figure 5g), but no tumour deposits were seen. Moreover, the pulmonary metastases formed primarily nodular deposits (Figure 5h) which were usually smaller, and the percentage of micrometastases in blood vessels was 87%, significantly higher ($\chi^2$ test; $P<0.01$) than obtained from s.c. tumours growing in intact rats.

**Table V** Histological and immunocytochemical staining patterns of cells derived from SMT-2A tumours

| Reagent | *Cells*[a] Rat mammary-land gland | Rama 900 | Rama 950 |
|---|---|---|---|
| Anti-MFGM | +[b] | − | − |
| Anti-actin/myosin | +[c] | − | − |
| Anti-keratin | +[c] | − | ± |
| Anti-laminin/type IV collagen | +[c] | − | + |
| Anti-factor VIII | − | − | − |
| Non-specific esterase | − | − | ± |
| Hoechst 33258-Giemsa on mouse ascites | − | −[d] | +[e] |

[a]Staining was graded as follows: −, no detectable staining; ±, 5–10% of the cells showed positive staining; +, greater than 20% of the cells showed positive staining. Grading of only the parenchymal tissue is recorded (**Materials and methods**); [b]Only the epithelial cells stain; [c]Only the myoepithelial cells stain; [d]No staining of the nuclei of loosely-adherent cells from mouse ascites; [e]Greater than 90% of nuclei positive for the adherent cells from mouse ascites, but none were positive for the adherent cells from rat ascites.

**Figure 6** Immunocytochemical and histochemical staining of SMT-2A cells. (**A**) Immunocytochemical staining of sections of collagen gel cultures of Rama 950 cells with antiserum to laminin showing intense cytoplasmic staining. Bar = 20 $\mu$m, ×410. (**B**) Hoechst 33258-Giemsa staining of a section of mouse liver showing intense clustering of stain in the chromatin. (**C**) Hoechst 33258-Giemsa staining of a section of rat liver showing little staining of the dispersed chromatin. (**D**) Hoechst 33258-Giemsa staining of a section of a primary tumour in a nude mouse induced by the s.c. injection of tumour cells from a nude mouse ascitic tumour. The larger tumour cells show little staining of the dispersed chromatin, while the host lymphocyte-like cells possess intensely staining nuclei (arrows). In **B** to **D** bars = 10 $\mu$m, ×825.

### *Immunocytochemical and enzymatic staining patterns of cultured cells and tumours*

Antiserum to MFGM, which selectively stains the epithelial cells, and antiserum to human keratin, which selectively stains the myoepithelial cells of the normal rat mammary gland (Warburton *et al.*, 1982) both failed to stain Rama 900 cells from cell pellets or in tumours. Antiserum to human keratin, but not that to MFGM stained 5–10% of the Rama 950 cells with a perinuclear distribution (Table V). Anti-

**Figure 7** Agarose gel electrophoretograms of isoenzymes present in extracts of SMT-2A cells. (**A**) Gel stained for lactate dehydrogenase and loaded with extracts from (a) Rama 900 cells grown in culture. (b) Rama 950 cells grown in culture. (c) Ascites cells taken directly from a nude mouse originally inoculated with Rama 900 cells. (d) Ascites cells from a nude mouse after two passages *in vitro*. The thick arrows indicate the position of the isoenzymes found in rat and mouse tissues. (**B**) Gel stained for purine nucleoside phosphorylase and loaded with extracts from (a) Rama 900 cells grown in culture. (b) Rama 950 cells grown in culture. (c) Loosely-adherent cell fraction from a nude mouse ascitic tumour. (d) Adherent cell fraction from a nude mouse ascitic tumour induced by Rama 900 cells. (e) Loosely-adherent cell fraction from a nude mouse ascitic tumour grown for two passages in culture. The thick arrows indicate the position of the isoenzyme found in rat and mouse tissues. The thin arrows indicate the direction of increasing mobility towards the cathode, and the original site of application of each sample on the gel is designated by 'O'.

serum to actin or myosin, which preferentially stains the myoepithelial and smooth muscle cells in rat mammary glands, failed to stain either cell type, but antiserum to the basement membrane proteins laminin or type IV collagen stained Rama 950 (Figure 6a), but not Rama 900 cells (Table V). Anti-factor VIII serum, which stains endothelial cells (Kiyoshi *et al*., 1980), failed to stain either cell type, but non-specific esterase (Davis & Ornstein, 1959), which stained histiocytes and macrophages in rat mammary glands, stained 5–10% of the adherent cell fraction from ascitic tumours (Table V). There was no change in any of the staining patterns when the more metastatic Rama 900-17 cells were treated directly or when growing as tumours in syngeneic rats (not shown).

*Origin of cells from mouse ascites tumours*

Loosely-adherent and adherent cells from the ascitic fluid of mice given i.p. injections of Rama 900 cells were morphologically and ultrastructurally identical to Rama 900 and Rama 950 cells, respectively (Figure 1). The loosely-adherent cells were highly aneuploid with an average chromosomal number ($\pm$s.e.) of $91 \pm 7$, similar to the values of $76 \pm 10$ and $86 \pm 6$ for early passage (number 8) and late passage (number 22) Rama 900 cells, respectively. The adherent cells from mouse ascites possessed the normal complement of mouse chromosomes of $40 \pm 1$, compared with the normal rat complement of $42 \pm 1$ for Rama 950 cells. Hoechst 33258-Giemsa staining showed intense clustering of stain in the chromatin of most mouse liver cells (Figure 6b), but not in rat liver cells (Figure 6c). The majority of the adherent cells from mouse ascites also showed clumps of heavily stained chromatin, but this staining was not observed in the loosely-adherent cells from mouse ascites (Table V) nor in the tumour cells of solid tumours growing in nude mice (Figure 6d).

Separation of the isoenzymes of LDH on agarose gels showed that rat liver possessed two slower running and mouse liver two faster running components (Everse & Kaplan, 1973). Before injection into nude mice Rama 900 cells possessed the two rat isoenzymes, but the resultant mouse ascites cells contained one and after subculture the two faster running mouse isoenzymes as well (Figure 7a). PNP consisted of one slower and one faster running isoenzyme for rat and mouse liver, respectively. Before injection into nude mice Rama 900 cells contained only the rat isoenzyme. After injection the loosely-adherent cells from the mouse ascites mainly contained the rat enzyme, whilst the adherent cells predominantly contained the mouse enzyme (Figure 7b).

### Discussion

Culturing the SMT-2A ascites tumours from syngeneic rats yields at least two discrete cell types: the loosely-adherent Rama 900 cells and the adherent Rama 950 cells.

The loosely-adherent Rama 900 cells represent the neoplastic cells, since they alone are aneuploid, and produce malignant tumours when re-introduced into syngeneic rats (Figure 1). Their cellular and ultrastructural morphologies and immunocytochemical staining patterns are identical to the neoplastic cells of the original SMT-2A solid tumour (Dunnington *et al*., 1984*a*). The dependence of Rama 900 tumour cells on a specific feeder layer of Rama 950 cells for growth *in vitro*, and for a shorter time before the rats become moribund, probably reflects a nutritional and/or growth requirement of the Rama 900 cells (Miller *et al*., 1980). These properties are not unique to a single variant of SMT-2A tumour cells, since primary cultures of the parental SMT-2A ascites tumours repeatedly yield the same two morphological cell types, and the loosely-adherent type is always dependent on the adherent type for growth in culture and *in vivo* (unpublished results). The incidence of tumours and the sites of metastases of the Rama 900 cell strain in syngeneic rats are also similar to those of the original SMT-2A solid tumour (Kim, 1979). The more extensive metastasis in syngeneic rats of Rama 900 cells at passage 17 than at passage 11 may be associated with the development of autonomous growth *in vitro* at about passage 19 (Figure 1). However, with this one exception, our methods of culturing do not appear to have dramatically affected the tumorigenic or metastasising ability of the original tumour cells.

The Rama 950 adherent feeder cells are non-neoplastic host cells, and the presence of intermediate filaments, microvilli and basement membrane proteins in the majority and the lack of staining with anti-factor VIII serum (Kiyoshi *et al.*, 1980) or for non-specific esterase (Leder, 1967) distinguishes them from fibroblasts, endothelial cells or macrophages. However, the epithelial-like features of Rama 950 cells are more akin to those of mesothelial cells. Thus, in most mesothelial cells the keratin intermediate filaments are scanty and are usually found round the nucleus, the microvilli are much longer and slender with length to diameter ratios ranging from 10 to 40 (Bewtra & Greer, 1985), banded type I collagen is also seen *in vitro* (Harvey & Amlot, 1983) as well as type IV basement membrane collagen *in vivo* (Whitaker, 1977), the intercellular junctions are usually incomplete and pinocytotic vesicles are also present (Bewtra & Greer, 1985). The gradual change of polygonal, mesothelial cells to spindle-cell forms on sub-culture may reflect different histological types of mesothelial cell seen *in vivo* (Bolen & Thorning, 1980) and/or degenerative changes *in vitro* (McGowan & Bunnag, 1974). The fact that s.c. injection of Rama 950 feeders with Rama 900 cells produces a shorter time before the rats become moribund, and hence a possible increase in the rate of metastasis, suggests that such mesothelial cells may also exert a 'feeder' effect at this site, but not at the other two sites *in vivo*. Variation of metastatic potential with site of implantation has also been observed in other systems (Meyvisch, 1983; Unemori *et al.*, 1984).

The results of our experiments in thymectomised rats suggest that the tumorigenic and metastasising properties of Rama 900 cells are suppressed in these animals, implying a possible direct or indirect role of the thymus in malignancy of this cell strain. The results of experiments in nude mice are more difficult to interpret since it is not entirely clear whether the differences that arise between intact rats and nude mice are due to differences in species (Gershwin *et al.*, 1982) or to the absence of the thymus (Kim, 1984). However, they are largely in agreement with those of Kim (1984), who showed that many of the transplanted SMT-2A tumours were eventually rejected by nude mice, and no metastases were seen during the subsequent two months. In the present study we have extended this observation period to 140 days and find that metastases do eventually occur, but their incidence is always less than that seen in the intact rat. In particular no lymph node metastases are seen in the nude mice, whereas metastasis to the lymph nodes invariably occur in rats bearing Rama 900 or SMT-2A tumours (Kim, 1979). Furthermore, the pattern of micrometastases in the lungs of nude mice is more consistent with haematogenous spread, whereas that in intact rats from s.c. primary sites is more consistent with lymphatic permeation (Kim, 1979). These results are not due to simple anatomical differences between rats and mice, since the results are maintained when cells are introduced s.c. into the interscapular fat pad of both rodents.

The isoenzyme/Hoechst 33258-Giemsa staining pattern, chromosomal complement and ultrastructure of the loosely-adherent ascites cells from nude mice injected i.p. with Rama 900 cells demonstrate that these cells are derived from Rama 900 cells, rather than from cells of spontaneous tumours of mouse origin. Thus ascites passage in nude mice has probably selected variants (Poste *et al.*, 1981; Nicholson *et al.*, 1983) from the original Rama 900 cell population lacking tumorigenic ability in their original rat host, but which now demonstrate increased metastatic properties in the nude mouse environment (Figure 1). Alterations of the *in vivo* properties of tumour cells may also be important in studies involving xenografts of human tumours into nude mice.

We thank Miss Christine Hughes, Mrs Anna Twiston Davies and Mrs Nina Perusinghe for expert technical assistance, Ms Linda Lovell for expert animal care, and Dr M.J. Warburton for gifts of antisera. This work was supported by the Ludwig Institute for Cancer Research, the Cancer and Polio Research Fund, and the Cancer Research Campaign, UK.

### References

AZZOPARDI, J.J. (1979). *Problems in Breast Pathology*. W.B. Saunders: Philadelphia.

BAUM, M. (1978). The curability of breast cancer. In *Breast Management – Early and Late*, Stoll, B. (ed.) p. 3. Heinemann: London.

BENNETT, D.C., PEACHEY, L.A., DURBIN, H. & RUDLAND, P.S. (1978). A possible mammary stem cell line. *Cell*, **15**, 283.

BEWTRA, C. & GREER, K.P. (1984). Ultrastructural studies of cells in body cavity effusions. *Acta Cytol.*, **29**, 226.

BOLEN, J.W. & THORNING, D. (1980). Mesotheliomas: a light and electron microscopic study concerning histogenetic relationships between the epithelial and mesenchymal variants. *Am. J. Surg. Pathol.*, **4**, 451.

CARR, I. (1983). Lymphatic metastasis. *Cancer Metastasis Rev.*, **2**, 307.

DAVIS, B.J. & ORNSTEIN, L. (1959). High resolution enzyme location with a new diazo reagent, hexazonium pararosanalin. *J. Histochem. Cytochem.*, **7**, 297.

DUNNINGTON, D.J., KIM, U., HUGHES, C.M., MONAGHAN, P., ORMEROD, J. & RUDLAND, P.S. (1984*a*). Loss of myoepithelial cell characteristics in metastasizing rat mammary tumors relative to their nonmetastasizing counterparts. *J. Natl Cancer Inst.*, **72**, 455.

DUNNINGTON, D.J., KIM, U., HUGHES, C.M., MONAGHAN, P. & RUDLAND, P.S. (1984*b*). Lack of production of myoepithelial variants by cloned epithelial cell lines derived from the TMT-081 metastasizing rat mammary tumor. *Cancer Res.*, **44**, 5338.

EVERSE, J. & KAPLAN, N.O. (1973). Lactate dehydrogenases: structure and function. *Adv. Enzymol.*, **37**, 61.

GERSHWIN, M.E., RUEBNER, B.H. & IKEDA, R.M. (1982). Transplantation and metastasis of NB prostate neoplasia in congenitally athymic (nude) mice, nude mice treated with antilymphocyte sera and congenitally athymic rats. *Exp. Cell Biol.*, **50**, 145.

GHOSH, S.K., GROSSBERG, A.L., KIM, U. & PRESSMAN, D. (1979). A tumor-associated, organ-specific antigen characteristic of spontaneously metastatic rat mammary carcinoma. *J. Natl Cancer Inst.*, **62**, 1229.

GHOSH, S., ROHOLT, O.A. & KIM, U. (1983). Establishment of two nonmetastasizing and one metastasizing rat mammary carcinoma cell lines. *In Vitro*, **19**, 919.

GILBERT, H.A. & KAGAN, A.R. (1976). Metastasis, incidence, detection and evaluation without histologic confirmation. In *Fundamental Aspects of Metastasis*, Weiss, L. (ed). p. 385. Elsevier/ North Holland Biomedical Press: Amsterdam.

GIOVANELLA, B.C., STEHLIN, J.S., WILLIAMS, L.J., LEE, S.-S. & SEPHARD, R.C. (1978). Hetero-transplantation of human cancers in nude mice. A model system for human cancer chemotherapy. *Cancer*, **42**, 2269.

GUSTERSON, B.A., WARBURTON, M.J., MITCHELL, D., ELLISON, M., NEVILLE, A.M. & RUDLAND, P.S. (1982). Distribution of myoepithelial cells and basement membrane proteins in the normal breast and in benign and malignant breast disease. *Cancer Res.*, **42**, 4763.

HARVEY, W. & AMLOT, P.L. (1983). Collagen production by human mesothelial cells *in vitro*. *J. Pathol.*, **139**, 337.

KIM, U. (1979). Factors influencing metastasis of breast cancer. In *Breast Cancer – Advances in Research and Treatment*, Vol. 3, McGuire, W.L. (ed) p. 1. Plenum Press: New York.

KIM, U. (1984). On the immunogenicity of tumor cells and the pattern of metastasis. In *Cancer Invasion and Metastasis: Biologic and Therapeutic Aspects*, Nicholson, G.L. & Milas, L. (ed) p. 337. Raven Press: New York.

KIYOSHI, M., ROSAI, J. & BURGDOPRF, W.H.C. (1980). Localization of factor VIII-related antigens in vascular endothelial cells using immunoperoxidase method. *Am. J. Surg. Pathol.*, **4**, 273.

LEDER, L.D. (1967). *Der Blutmonocyt*. Springer-Verlag: Berlin.

McGOWAN, L. & BUNNAG, B. (1974). Morphology of mesothelial cells in peritoneal fluid from normal women. *Acta Cytol.*, **18**, 205.

MEYVISCH, C., (1983). Influence of implantation site on formation of metastases. *Cancer Metastasis Rev.*, **2**, 295.

MILLER, B.E., MILLER, F.R., LEITH, J. & HEPPNER, G.H. (1980). Growth interaction *in vivo* between tumor subpopulations derived from a single mouse mammary tumor. *Cancer Res.*, **40**, 3977.

NERI, A., WELCH, D., KAWAGICH, T. & NICHOLSON, G.L. (1982). Development and biologic properties of malignant cell sublines and clones of a spontaneously metastasizing rat mammary adenocarcinoma. *J. Natl Cancer Inst.*, **68**, 507.

NICHOLSON, G.L., STECK, P.A., WELCH, D.R. & LEMBO, T. (1983). Heterogeneity and instability of phenotypic and metastatic properties of local tumor – and metastasis-derived clones of a mammary adenocarcinoma. In *Understanding Breast Cancer: Clinical and Laboratory Concepts*, Rich, M., Hager, J. & Furmanski, P. (eds) p. 145. M. Dekker: New York.

ORMEROD, E.J. & RUDLAND, P.S. (1982). Mammary gland morphogenesis *in vitro*: formation of branched tubules in collagen gels by cloned rat mammary cell line. *Develop. Biol.*, **91**, 360.

POSTE, G., DOLL, J. & FIDLER, I.J. (1981). Interactions among clonal subpopulations affect stability of the metastatic phenotype in polyclonal populations of B16 melanoma cells. *Proc. Natl Acad. Sci. USA.*, **78**, 6226.

ROTHFELS, K.H. & SIMINOVITCH, L. (1958). An air-drying technique for flattening chromosomes in mammalian chromosomes grown *in vitro*. *Stain Technol.*, **33**, 73.

RUDLAND, P.S., GUSTERSON, B., HUGHES, C.M., ORMEROD, E.J. & WARBURTON, M.J. (1982). A neoplastic rat mammary stem cell line generates two forms of tumors in nude mice. *Cancer Res.*, **42**, 5196.

SORDAT, B., TAMAOKI, N. & POVLSEN, C.O. (1977). List of Human tumors transplanted to nude mice. In *Proc. Second Int. Workshop on Nude Mice*, Normura, T., Ohawa, N., Tamaoki, N. & Fujiwara, K. (eds) p. 586, University of Tokyo Press: Tokyo.

UNEMORI, E.N., WAYS, N. & PITELKA, D.R. (1984). Metastasis of murine mammary tumour lines from the mammary gland and ectopic sites. *Br. J. Cancer*, **49**, 603.

WARBURTON, M.J., MITCHELL, D., ORMEROD, E.J. & RUDLAND, P.S. (1982). Distribution of myoepithelial cells and basement membrane proteins in the resting, pregnant, lactating and involuting rat mammary gland. *J. Histochem. Cytochem.*, **30**, 667.

WHITAKER, D. (1977). Cell aggregates in malignant mesothelioma. *Acta Cytol.*, **21**, 236.

WILLIAMS, J.C., GUSTERSON, B.A., MONAGHAN, P., COOMBES, R.C. & RUDLAND, P.S. (1985). Isolation and characterization of clonal cell lines from transplantable metastasizing rat mammary tumor, TR2CL. *J. Natl Cancer Inst.*, **74**, 415.